# Colocalization Analysis of Lipo-Deoxyribozyme Consisting of DNA and Protic Catalysts in a Vesicle-Based Protocellular Membrane Investigated by Confocal Microscopy

**DOI:** 10.3390/life11121364

**Published:** 2021-12-08

**Authors:** Yuiko Hirata, Muneyuki Matsuo, Kensuke Kurihara, Kentaro Suzuki, Shigenori Nonaka, Tadashi Sugawara

**Affiliations:** 1 Department of Chemistry, Faculty of Science, Kanagawa University, Tsuchiya, Hiratsuka 259-1293, Kanagawa, Japan; yuikohirata@gmail.com; 2 Department of Chemistry, Graduate School of Integrated Sciences for Life, Hiroshima University, Kagamiyama, Higashi-Hiroshima 739-8526, Hiroshima, Japan; muneyuki@hiroshima-u.ac.jp; 3 Exploratory Research Center on Life and Living Systems (ExCELLS), Myodaiji, Okazaki 444-8787, Aichi, Japan; kkurihara2021@gmail.com (K.K.); snonaka@nibb.ac.jp (S.N.); 4 National Institute for Basic Biology, Myodaiji, Okazaki 444-8585, Aichi, Japan

**Keywords:** model protocell, giant vesicle, supramolecular catalyst, lipo-deoxyribozyme, colocalization analysis, confocal laser scanning fluorescence microscope

## Abstract

The linkage between the self-reproduction of compartments and the replication of DNA in a compartment is a crucial requirement for cellular life. In our giant vesicle (GV)-based model protocell, this linkage is achieved through the action of a supramolecular catalyst composed of membrane-intruded DNA and amphiphilic acid catalysts (**C@DNA**) in a GV membrane. In this study, we examined colocalization analysis for the formation of the supramolecular catalyst using a confocal laser scanning fluorescence microscope with high sensitivity and resolution. Red fluorescence spots emitted from DNA tagged with Texas Red (Texas Red-DNA) were observed in a GV membrane stained with phospholipid tagged with BODIPY (BODIPY-HPC). To our knowledge, this is the first direct observation of DNA embedded in a GV-based model protocellular membrane containing cationic lipids. Colocalization analysis based on a histogram of frequencies of “normalized mean deviation product” revealed that the frequencies of positively correlated [lipophilic catalyst tagged with BODIPY (BODIPY-**C**) and Texas Red-DNA] were significantly higher than those of [BODIPY-HPC and Texas Red-DNA]. This result demonstrates the spontaneous formation of **C@DNA** in the GV membrane, which serves as a lipo-deoxyribozyme for producing membrane lipids from its precursor.

## 1. Introduction

The intrinsic nature of a living system is characterized by self-reproduction and spontaneous generation of a functional supramolecular system that ensures robust self-reproduction over generations, enabling the possibility of eventual evolution [1,2,3,4]. To establish an “individual” as a living system, a compartment encapsulating information-containing substances, enzymes, and other molecules is indispensable [4,5]. Typically, a biological compartment is composed of a semipermeable membrane that passes substrates and inorganic ions and discards waste products in a selective manner for metabolic reactions [6,7]. Active transport selectivity is generally regulated by membrane proteins. Thus, the cellular membrane separates the inner reaction field from the outer reaction field. A primitive protocell that displays cooperative functions in a lipid membrane or communication between the outer and inner reaction fields through a lipid membrane should be constructed to study these processes [8,9,10,11,12,13].

**Figure 1:**
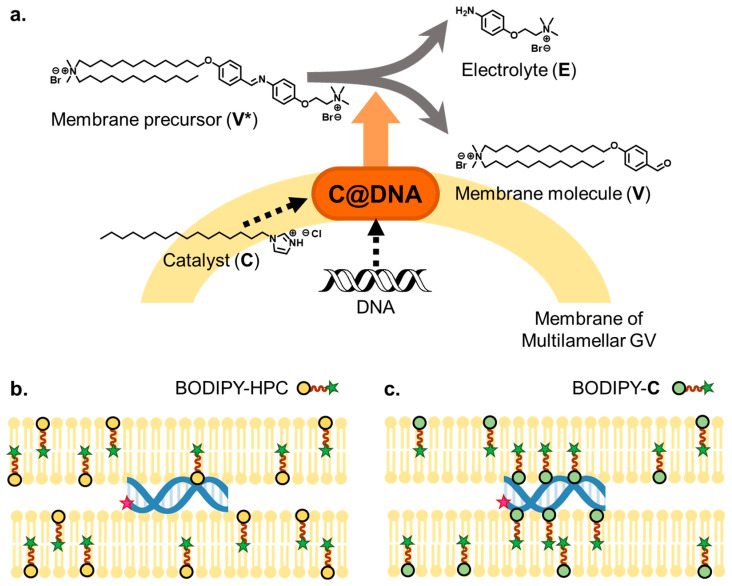
(**a**) Reaction scheme of membrane lipid production (**V**) by **C@DNA**. Hydrolysis of membrane lipid precursor (V*) produces membrane lipid **V** and electrolyte (**E**) in aid of the supramolecular catalyst **C@DNA**, which is a complex between catalyst **C** and DNA. **C** is a lipophilic and protic catalyst with a hydrophilic imidazolium bromide head group and a hydrophobic tail group. (**b**,**c**) Schematic representation of the distribution of DNA imbedded in the multilamellar GV membrane, depicted as red, and phospholipid (BODIPY-HPC) or amphiphilic catalyst **C** (BODIPY-**C**), depicted as green, in (**b**) or (**c**), respectively. DNA in a GV membrane lies in the water phase between the two bilayers. (**b**) Phospholipid (BODIPY-HPC) distributes randomly in bilayer membranes. (**c**) Amphiphilic catalysts (BODIPY-**C**) distributed in bilayer membranes are closer to the phosphate anions of DNA.

Giant vesicle (GV)-based model protocells (diameter > 1 µm) have gained attention for revealing the intrinsic nature of cellular life [14,15,16,17,18]. Because DNA replication in GVs and GV reproduction occur through independent dynamics, the linkage between the two dynamics is difficult to observe. However, in a previous study, a distinct linkage between DNA replication and multilamellar GV reproduction was achieved when DNA was amplified in GV containing cationic membrane lipids (**V**) and a precursor of lipid membrane was added to a GV dispersion. The mechanism of linked proliferation was predicted to involve amplified DNA penetrating a multilamellar GV membrane containing cationic lipids to form a local lipoplex [19]. The penetrated DNA gathered protic catalysts (**C**s), which were distributed in the GV membrane and formed a supramolecular catalyst named **C@DNA** (Figure 1a). This catalyst promoted the rapid and local production of membrane lipids from their precursors. As a result, the GV-based protocell deformed in a budded form and eventually divided into almost equivolume daughter GVs [20]. Although **C@DNA** does not manipulate DNA, it catalyzes the hydrolysis of V* by cleaving the imine bond. This catalytic behavior is somewhat similar to the action of ribozyme; RNA forms a complex with a metal ion and can catalyze the hydrolysis of RNA and other substrates [21,22]. In this sense, the supramolecular catalytic **C@DNA** in which DNA is associated with the lipophilic catalyst **C** can be regarded as a “lipo-deoxyribozyme” [20].

The following evidence has been obtained for the intrinsic contribution of **C@DNA** to enhancement of membrane lipid **V**-formation. When DNA was amplified in the GV containing cationic lipid **V** (10 mol%, relative amount in the membrane lipids) by PCR in the presence of SYBR Green I, a fluorescence indicator of DNA, intense green fluorescence, was observed in the GV membrane rather than in the inner water phase. This observation suggests that the PCR-amplified DNA as a polyanionic molecule intruded the multilamellar GV membrane containing cationic membrane lipids **V**s. In contrast, green fluorescence was not observed when the inner water phase of GV did not contain **V**, although we could not exclude the possibility that the amplified DNA adhered to the inner surface of the GV membrane [19].

To examine the protic catalyst **C** in the proximity of DNA, we conducted Förster-type resonance-energy-transfer experiments in a previous study and found that numerous protic catalysts were present near the DNA [20]. These results strongly suggest that a complex formed between DNA and protic catalyst **C**. However, it was difficult to conclude that the entire **C@DNA** was buried in the GV membrane because the energy-transfer distance in this mechanism was greater than 10 nm, which is longer than the width (~8 nm) of the bilayer membrane. For the catalytic effect of membrane lipid **V** formation from its precursor V*, 10-fold enhanced catalytic activity for the hydrolysis of V* was observed only when the GV membrane contained DNA and catalyst **C**. No acceleration was detected when the GV contained each of the two components. This result demonstrates that catalytic activity was enhanced by the cooperative interaction between DNA and the protic catalyst. The DNA length (374, 1164, 3200 base pairs (bp)) significantly influenced the self-proliferation of GV-based protocells under confocal laser scanning microscopy and flow cytometry [20]. Even the emergence of the predominant species of the GV containing 1164 bp DNA appeared through competition between GV-based protocells containing different lengths of DNA [10].

The formation of membrane lipid **V** in the GV membrane, assisted by **C@DNA**, slightly resembles the activity of membrane proteins, although phospholipid biosynthesis in a living cell proceeds in the membrane of the endoplasmic reticulum, which is an inner organelle [23,24,25]. In the present study, we evaluated the formation of **C@DNA** via direct observation of **C@DNA** in the GV membrane and colocalization analysis of catalyst **C** and embedded DNA using a confocal laser scanning microscope with high sensitivity and resolution (Figure 1b,c).

## 2. Materials and Methods

### 2.1. Materials

1-Palmitoyl-2-oleoyl-*sn*-glycero-3-phosphocholine (POPC) was purchased from NOF (Tokyo, Japan), and 1,2-distearoyl-*sn*-glycero-3-phosphoethanolamine-*N*-[methoxy(polyethyleneglycol)-1000] (DSPE-1K) was purchased from Nanocs, Inc. (New York, NY, USA). Cholesterol (chol) was purchased from Wako Pure Chemical Industries (Osaka, Japan). Membrane lipid molecule **V**, lipophilic catalyst **C**, catalyst **C** tagged with BODIPY (BODIPY-**C**), and membrane lipid precursor V* were synthesized, as previously reported [26,27]. The restriction enzyme EcoRI, pBR322 vector Wizard^®^ SV Gel, and PCR Clean-up system for purification were purchased from Promega Corporation (Madison, WI, USA). The primers were purchased from Sigma-Aldrich (St. Louis, MO, USA). A primer tagged with Texas Red (TR-primer) was purchased from Fasmac (Kanagawa, Japan). DNA ladder markers and DNase I were purchased from Takara Bio (Shiga, Japan). KOD-plus-DNA polymerase was purchased from Toyobo (Osaka, Japan). 2-(4,4-Difluoro-5,7-dimethyl-4-bora-3*a*,4*a*-diaza-*s*-indacene-3-dodecanoyl)-1-hexadecanoyl-*sn*-glycero-3-phosphocholine (BODIPY-HPC) was purchased from Thermo Fisher Scientific (Waltham, MA, USA).

### 2.2. Preparation of GV Containing PCR Reagents with and without Cationic Lipid **V**

Dispersed GVs and amplified DNA were prepared, as previously reported [18,19], with the compositions shown in Table 1, Table 2, Table 3 and Table 4. GV membranes with and without cationic lipid **V** were prepared separately, which encapsulated the PCR reagents. The fluorescence probes used were phospholipid or **C** tagged with BODIPY or DNA tagged with Texas Red (Texas Red-DNA), and the latter was synthesized in situ during PCR using primers tagged with Texas Red.

### 2.3. Confocal Observation of DNA Tagged with Texas Red in GV with and without Cationic Membrane Lipid **V**

Dispersions of GVs with and without cationic membrane lipid **V** were prepared. The GV membranes of both types of GVs were stained with BODIPY-HPC or BODIPY-**C** and contained 1164-bp DNA tagged with Texas Red (0.14 mM). The incubated dispersion was divided into PCR tubes (100 µL) and placed in a thermal cycler to amplify the DNA (94 °C for 60 s, 68 °C for 90 s) for 20 cycles. Microscopic observation of the PCR-subjected GVs was conducted at more than 24 h after PCR. Confocal fluorescence microscopy was conducted on PCR-subjected GVs containing cationic membrane lipid **V** with 2048 × 2048 active pixels using two separate channels: a BODIPY channel (488 nm excitation/505–540 nm emission) and a Texas Red channel (555 nm excitation/600–750 nm emission) under the same gain, exposure time and laser intensity.

### 2.4. Colocalization Analysis Method

Confocal microscope images recorded using the BODIPY and Texas Red channels were output as TIFF using Leica LASX microscope measurement software (ver. 3.7.1.21655; Wetzlar, Germany). The image file output was read using Fiji image-analysis software (ver. 1.53) [28]. Noise in the images recorded through the BODIPY and Texas Red channels was reduced as follows. The threshold values of BODIPY/Texas Red were 40/30 and 20/30 for the heatmaps and histograms, respectively. A plug-in of the colocalization heatmap was used with the obtained images to calculate the normal mean derivation product (nMDP) for each pixel.

If the fluorescence intensities of two types of probe-tagged substrates in a pixel, which is the smallest colored digital unit with a size of 90.14 × 90.14 nm^2^ of this instrument, are both high, the pixel is recognized as positive (if both intensities are low, a pixel is also positive) by the image-analysis software (Fiji). These positive pixels are shown as warm colors (yellow-red) in a heatmap. In contrast, if the fluorescence intensity emitted from one of the two substances is strong and the other is weak, a pixel is recognized as negative, and these pixels are shown as a cool color (greenish-blue, blue) in a heatmap. Thus, a heatmap shows the positive and negative correlations in pixels to visualize the colocalization of two substances tagged with different probes.

Quantitative analysis was conducted using Fiji software. An index of the colocalization of two substances within a pixel was expressed as the nMDP, defined by Equation (1) [29,30,31,32].

(1) $$\mathrm{nMDP}=\frac{\left(BD_{i}-BD_{\mathrm{av}\;}\right)\left(TR_{i}-TR_{\mathrm{av}\;}\right)}{\left(BD_{\max}-BD_{\mathrm{av}\;}\right)\left(TR_{\max}-TR_{\mathrm{av}\;}\right)}\;$$

where *BD*_i_ represents the fluorescence intensity for a given pixel (i) in the image emitted from BODIPY, *BD*_av_ represents the average fluorescence intensity of all pixels, and *BD*_max_ represents the maximum fluorescence intensity of all pixels. *TR*_i_, *TR*_av_, and *TR*_max_ have the same meaning as in BD cases; however, the images are emitted from Texas Red.

## 3. Results

### 3.1. Observation of DNA in GV Membrane Containing **V** by Confocal Microscopy

According to previous studies [20], the penetration of DNA, an anionic polymer, into a GV membrane is achieved via the hydrophobic covering on its surface by cationic membrane lipid **V**s. We examined DNA penetration into a GV membrane containing or not containing the cationic membrane lipid **V** using confocal laser scanning fluorescence microscopy with catalyst **C** tagged with a fluorescence probe (BODIPY-**C**, energy donor) and DNA tagged with Texas Red (Texas Red-DNA, energy acceptor). The confocal laser scanning fluorescence microscope had a resolution of 250 nm along the x and y axes and 1 µm along the *z*-axis. Two-dimensional sliced images were obtained using confocal microscopy. To observe DNA embedded in a GV membrane containing cationic lipid **V** using confocal scanning laser microscopy, the GV membrane stained with BODIPY-HPC and Texas Red-DNA was prepared in situ during PCR amplification using template DNA tagged with Texas Red at the 5′-terminus (Figure S1). After this amplification step, the GV was incubated for at least 24 h to allow sufficient DNA penetration into the GV membrane.

**Figure 2:**
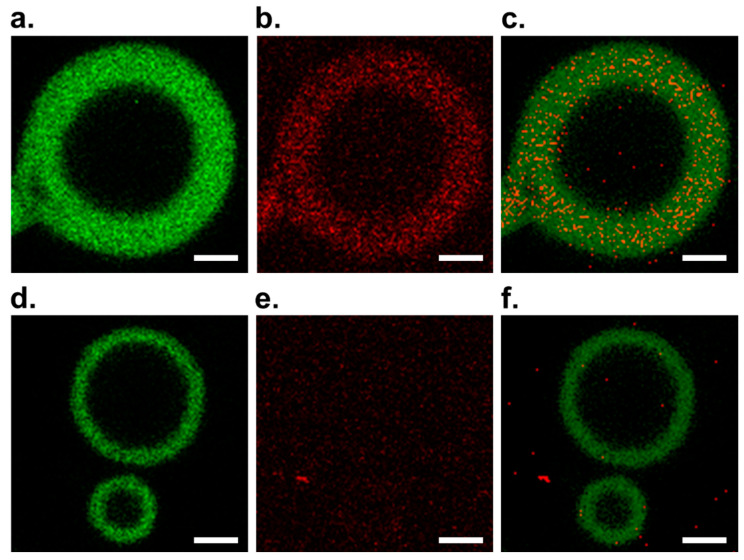
Confocal fluorescence microscope images obtained from PCR-subjected multilamellar GV containing cationic **V** in the upper row (**a–c**) and images obtained from PCR-subjected GV without cationic **V** in the lower row (**d–f**). (**a**) Image obtained through a BODIPY channel; (**b**) image obtained through a Texas Red channel; (**c**) overlapped image of images (**a**,**b**); (**d**) image obtained through a BODIPY channel; (**e**) image obtained through a Texas Red channel; (**f**) overlapped images (**d**,**e**). The images were edited using the same protocol and threshold for each channel. Scale bars represent 2 μm.

**Figure 3:**
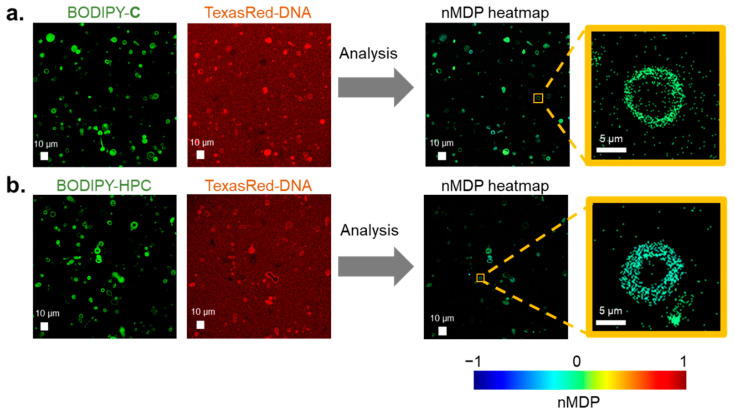
Confocal fluorescence microscope images emitted from BODIPY-**C** and Texas Red-DNA vs. BODIPY-HPC and Texas Red-DNA and their heatmaps drawn using colocalization analysis with nMDP values. (**a**) Fluorescence images of BODIPY-**C** and Texas Red-DNA and their heatmap using nMDP values; (**b**) fluorescence images of of BODIPY-HCP and Texas Red-DNA and their heatmap using nMDP values.

We acquired confocal fluorescence images of PCR-subjected multilamellar GVs and found a distinct difference in images between GVs with **V** (Figure 2a–c) and those without **V** (Figure 2d–f). Bright green fluorescence with small black voids emitted from the PCR-subjected GV membrane stained by BODIPY-HPC was observed through the BODIPY channel (Figure 2a). Figure 2b shows an image observed through the Texas Red channel, with red spots emitted from Texas Red-DNA in the GV membrane. Overlapping these two images via two individual channels produced Figure 3c, indicating that red fluorescence spots were emitted from Texas Red DNA in the GV membrane stained with BODIPY-HPC. In contrast, the confocal image of GV without **V** showed no localization of red fluorescence spots in the GV membrane (Figure 2e). These results strongly suggest that the presence of cationic lipid **V** in the GV membrane is indispensable for penetration of amplified DNA into the membrane.

### 3.2. Elucidation of the Formation of **C@DNA** in GV Membrane Using Colocalization Analysis

To obtain direct evidence for the construction of **C@DNA** in a GV membrane, we performed colocalization analysis of the DNA and catalyst **C** in GV membranes containing **V**. This quantitative method reveals whether two components stained using two different fluorescence probes exist in proximity to one another. For reliable colocalization analysis, accidental access between two components, such as the protic catalyst and phospholipids, must be avoided. The concentrations of BODIPY-**C** and BODIPY-HPC were diluted to 5 × 10^−3^ mol%. The brightness of the fluorescence intensities of BODIPY-HPC and BODIPY-**C** should be practically the same (Figure S2 and Table S1) in the confocal laser scanning microscopy images. Leakage of fluorescent light from a probe into a detecting channel of the fluorescence light emitted from a different probe must be prevented, and saturation of the detected fluorescence light from the probe must be avoided. These requirements were satisfied in previous experiments, as described in Figures S3 and S4.

GV membranes containing BODIPY-**C** (5 × 10^−3^ mol%) and BODIPY-HPC (5 × 10^−3^ mol%) were prepared. Confocal microscope images of GVs containing [BODIPY-**C** and Texas Red-DNA], together with [BODIPY-HPC and Texas Red-DNA], were recorded at five sites of 185 × 185 μm^2^, corresponding to 2048 pixels^2^, in a frame-seal chamber and examined using colocalization analysis (Figure 3). Because most of the DNA was incorporated into the GV membrane, the intensity of Texas Red-DNA in the aqueous phase was quite low. Hence, we restricted the colocalization analysis of [BODIPY-**C** and DNA] in reference to [BODIPY-HPC and Texas Red-DNA] to the GV membrane.

Confocal fluorescence microscopy images of [BODIPY-**C** and Texas Red-DNA] are shown in Figure 3 (left), and their fluorescence intensities in pixels were analyzed using Fiji software. The detailed method used for colocalization analysis and evaluation of the nMDP are described in Section 2.4 [29,30,31]. Heatmaps drawn using nMDP values show a relatively warm yellowish-green color (Figure 3a, right). In contrast, a heatmap derived from fluorescence images from [BODIPY-HPC and Texas Red-DNA] (Figure 3b, left) exhibited a cool blue color (Figure 3b, right). These two heatmaps suggest that the positive correlation between [BODIPY-**C** and Texas Red-DNA] was appreciably higher than that between [BODIPY-HPC and Texas Red-DNA].

**Figure 4:**
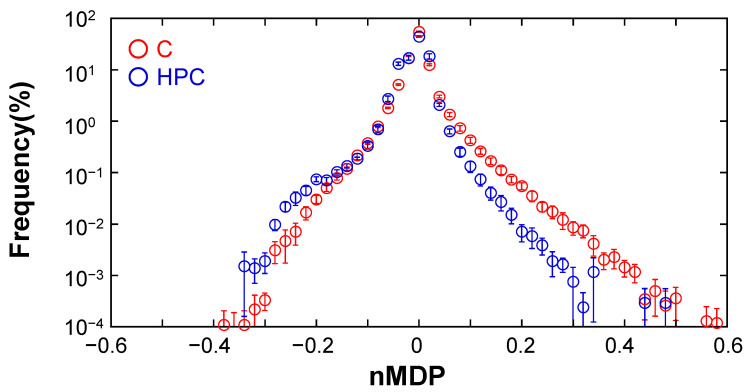
Histograms of nMDP of correlations in “DNA-Texas Red and BODIPY-**C**” and “DNA-Texas Red and BODIPY-HPC” (red open circle: BODIPY-**C**, blue open circle: BODIPY-HPC). The vertical axis shows the frequency of relative values of the averaged sums of pixels with nMDP values counted from five selected areas in the fields of view of PCR-subjected GVs. The horizontal axis represents the class of nMDP with a width of 0.02 over a range from −1 to 1. Error bar represents standard error derived from the five samples.

To analyze the colocalization of [BODIPY-**C** and Texas Red-DNA] compared to [BODIPY-HPC and Texas Red-DNA], a histogram obtained from the heatmaps of nMDP was drawn, as shown in Figure 4. The histogram was obtained from five fields of view (Figure S5, Tables S2 and S3). If a histogram is symmetrical with respect to the vertical axis, the ratio between the total frequencies of pixels with nMDP values higher than 0.1 and those with values lower than −0.1 should be 1. However, the obtained histogram of nMDP shows an asymmetrical shape on the vertical axis. The plot of the GV membrane containing [BODIPY-**C** and Texas Red-DNA] shows distinct asymmetry to the vertical axis: it is upshifted compared to that of [BODIPY-HPC and Texas Red-DNA] on the positive side. The frequencies of nMDP in the regions of more than +0.1 and less than −0.1 were calculated as 1.20 ± 0.17% and 0.53 ± 0.07%, respectively, against all analyzed pixels. The ratio of the sum of frequencies of the higher nMDP to the sum of the lower nMDP was 2.26 ± 0.17. This ratio indicates that the [BODIPY-**C** and Texas Red-DNA] frequency in the positive region was significantly higher than that in the negative region. In contrast, for GVs containing [BODIPY-HPC and Texas Red-DNA], the frequencies of nMDP in the same regions of more than +0.1% and less than −0.1 were 0.31 ± 0.09% and 0.68 ± 0.07%, respectively, and their ratio was 0.46 ± 0.31. Therefore, the negative correlation between BODIPY-HPC and Texas Red-DNA was slightly dominant over the positive correlation. In addition, the histogram of [BODIPY-HPC and Texas Red-DNA] shows a slight upward shift in the lower range compared to the approximately −0.2 observed in the negative region. This is because in a GV membrane, the protic catalyst **C** exists near the phosphate group of DNA because of the strong coulombic interaction. Once **C** occupies a site near DNA, HPC avoids this site because the head group of the zwitter-ionic phosphocholine is a quaternary ammonium.

Colocalization analysis demonstrates a positive correlation between protic catalyst **C** and DNA embedded in the GV membrane and weak negative correlation between the two ionic phospholipids (HPC) and DNA. This result demonstrates the higher possibility of protic catalyst **C** waiting in proximity to DNA embedded in the GV membrane containing cationic lipid **V** compared to a zwitter-ionic phospholipid. This strongly supports assessment that a supramolecular catalyst, **C@DNA**, was formed in the GV membrane, which functions as a lipo-deoxyribozyme in the production of membrane lipid **V** from its precursor V*.

## 4. Discussion

Red fluorescent spots emitted from Texas Red-DNA in the GV membrane were observed only in the GV membrane containing cationic lipid **V**. To the best of our knowledge, this is the first direct observation of membrane-intruded DNA that was amplified in the water phase of a GV-based protocell and provides strong evidence that cationic lipid V is necessary for DNA incorporation into the GV membrane.

**Figure 5:**
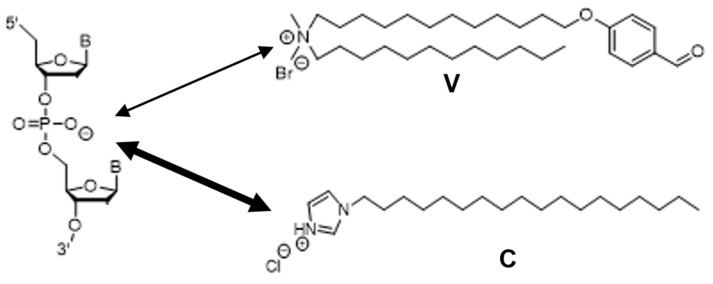
Electrostatic interaction between DNA as an anionic polymer and cationic membrane **V** or protic catalyst **C**.

The difference in the attractive interaction between DNA and cationic membrane lipid **V** and protic catalyst **C** can be explained as follows. Amplified DNA in the inner water phase of a GV by PCR can diffuse close to the inner surface of the GV membrane. DNA, which is located on the inner membrane surface, may be captured and covered by cationic lipid **V**s present in the GV membrane through ionic interactions, forming a local lipoplex (Figure 5). Inside the GV membrane, exchange between cationic **V** and protic catalyst **C** occurs because catalyst **C** is an imidazolium salt and a more naked cation than the tetra (ammonium) salt of **V**.

For DNA conformation in a GV membrane, the conformational conversion of DNA, such as the coil-globule transition, has been discussed extensively. The chain length of DNA (1164 bp) is estimated to be approximately 400 nm; however, it exists as a random coil with a persistence length of approximately 50 nm in the GV membrane, suggesting that the practical diameter is around 250 nm. Because the pixel size of a confocal laser scanning microscope is 90.14 × 90.14 nm^2^, whole DNA (1164 bp) cannot be detected in one pixel. However, the sizes of the corresponding fluorescence probes (Texas Red tagged with DNA and BODIPY tagged with phospholipid) were less than 2 nm. Hence, these fluorescence probes may reside together in the same pixel.

In addition, we found that the positive correlation between BODIPY-**C** and Texas Red-DNA was higher than that between BODIPY-HPC and Texas Red-DNA. This is expected, as the hydrophilic part of the phospholipid is a choline group with a zwitterion, whereas that of protic catalyst **C** is an imidazolium salt existing as a loose ion pair or free cation. Under the present conditions, energy-derived Förster resonance energy transfer from the excited BODIPY to TexasRed possibly occur and reduce the nMDP at the molecular level. However, against the nMDP reduction effect, the nMDP of [BODIPY-**C** and Texas Red-DNA] was sufficiently high. Hence, **C** interacts with the phosphate groups of Texas Red-DNA via coulombic interactions, forming a tight coulombic complex.

The division mechanism of our GV-based model protocell appears to be too simple compared to that of a contemporary living cell. However, many modern bacteria retain the ability to switch back to a wall-free state known as the L-form. Proliferation of the L-form does not require division machinery, and its division occurs via activation of a lipid-forming enzyme; the increased surface area-to-volume ratio destabilizes the elongated cell to divide. Our model protocell is an appropriate model for this type of division performed by modern bacteria [32,33]. Although our model protocell is not equipped with sophisticated molecular machinery, such as FtsZ, this model protocell is equipped with a preliminary division system that depends on the distribution of **C@DNA**. The distribution of **C@DNA** in the membrane may influence the morphological changes of the model protocell. Namely, after a certain incubation period, numerous **C@DNA**s are formed in the GV membrane, and membrane lipid **V** is produced around **C@DNA**. If a certain zone in the GV membrane is surrounded by multiple **C@DNA**s, the lateral compressibility caused by accumulated **V** within the zone becomes sufficiently high because the diffusional escape of **V** from the zone is suppressed by surrounding **C@DNA**s as a barrier to diffusional escape. A high lateral compressibility in the zone causes buckling of the membrane to generate budding deformation as a kinetically controlled structure. Interestingly, quite a few red fluorescence spots emitted from Texas Red-DNA appeared in the GV membrane, as shown in Figure 2c, but under these experimental conditions, membrane lipid precursor V* was not supplied. When V* is added under such a condition, it is expected that GVs containing numbers of **C@DNA** deforms into a budded form and divided into two GVs.

In conclusion, we showed that amplified DNA penetrated the GV membrane only when the GV membrane contained cationic membrane lipid **V**. Furthermore, colocalization analysis using confocal microscopy images revealed the **C@DNA** formation, which is a preliminary form of membrane-protein, recognized as lipo-deoxyribozyme [20]. Colocalization analysis is typically used to evaluate the structures of biological systems, such asa complexation between antigens and antibodies. This experiment is a good example of the applicability of this technique for analysis of the structure of supramolecular functional self-assembly, which is difficult to analyze using spectroscopic or diffraction methods. However, as stated in the results section (Section 3.1), the resolution of a confocal laser scanning fluorescence microscope is limited to 250 nm along the x and y axes and 1 µm along the *z* axis, compared with super-resolution techniques. To reveal the dynamic correlation between **C@DNA** and self-reproduction dynamics, structured illumination methods (SIM) would be the best super-resolution technique for a future scope of this research [34].

## Figures and Tables

**Table 1:** Membrane composition of giant vesicle with cationic membrane lipids.

	Additives (mL)	Concentration (mM)	mol%
POPC	77.2	10	77.3
**V**	16.8	2.5	4.21
**C**	33.6	2.5	8.41
DSPE-1K	67.2	0.25	1.68
Chol.	33.6	2.5	8.41
BP-HPC/BP-C	1	0.1	0.01

**Table 2:** Membrane composition of giant vesicle without cationic membrane lipids.

	Additives (mL)	Concentration (mM)	mol%
POPC	77.2	10	80.68
**C**	33.6	2.5	8.778
DSPE-1K	67.2	0.25	1.756
Chol	33.6	2.5	8.778
BP-HPC	1	0.1	0.01

**Table 3:** Composition of PCR solution.

	Additives (mL)	Initial Concentration (mM)	Final Concentration (mM)
Water	348	-	-
KOD buff.	50	-	-
MgSO_4_ aq.	20	25	0.499
dNTPs aq.	40	8	0.319
TR-primer	14	10	0.140
Reverse-primer	14	10	0.140
Template DNA	5	10	0.050
KOD aq.	10	-	-

**Table 4:** Composition of a solution to be added to the outer water phase.

	Volume of Additives (µL)
Deionized Water	430
10×KOD-plus-Buffer	50
100 mM CaCl_2_ aq	5
1 U/µL DNase I	15

## Data Availability

Not applicable.

## Supplementary Materials

The following are available online at https://www.mdpi.com/article/10.3390/life11121364/s1, Figure S1: Molecular structures of fluorescence-labeled lipids and DNA, Figure S2: Fluorescence intensity histograms of GVs stained with BODIPY-HPC and BODIPY-**C**, Figure S3: Fluorescence compensation in confocal microscopy observation, Figure S4: Prevention of fluorescence saturation in confocal microscopy observation, Figure S5: nMDP heatmaps for DNA-Texas Red and BODIPY-C and DNA-Texas Red and BODIPY-HPC originating the histogram in Figure 4, Table S1: Indistinguishability of analyzed fluorescence intensities between BODIPY-HPC and BODIPY-**C** in current confocal microscopy observations, Table S2: Number of pixels calculated nMDP on each heat maps represented in Figure S5, Table S3: Population ratio of pixels with magnitude of nMDP > −1.0 or nMDP < +0.1 on the nMDP heat map.
