# Supplementary material for: Colocalization Analysis of Lipo-Deoxyribozyme Consisting of DNA and Protic Catalysts in a Vesicle-Based Protocellular Membrane Investigated by Confocal Microscopy
Source: Life (Basel). 2021 Dec 8;11(12):1364. doi: 10.3390/life11121364 (PMC8707093; doi:10.3390/life11121364)
Supplement: Supplementary file 1 [file life-11-01364-s001.zip › life-1454899-SI.pdf]

Supplementary Materials

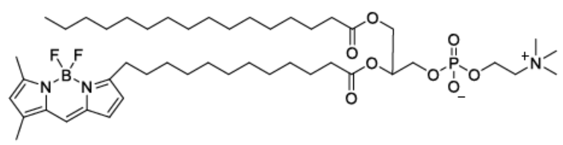

(a)

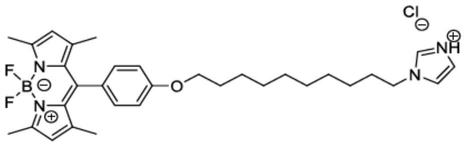

(b)

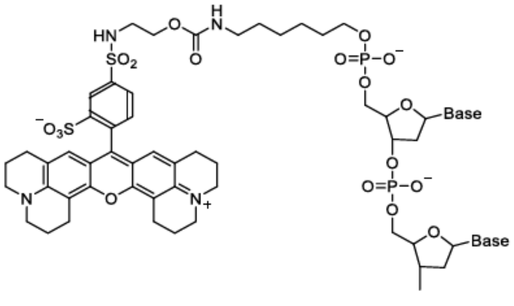

(c)

Figure S1. Molecular structures of fluorescence-labelled lipids and DNA. (**a**) BODIPY-HPC. (**b**)BODIPY-**C**. (**c**) Texas Red-DNA.

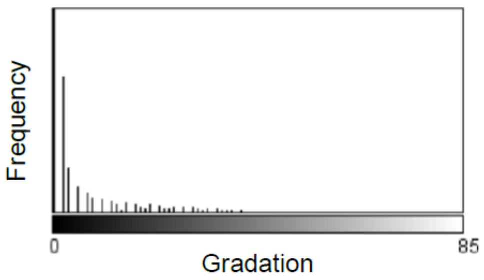

(**a**)

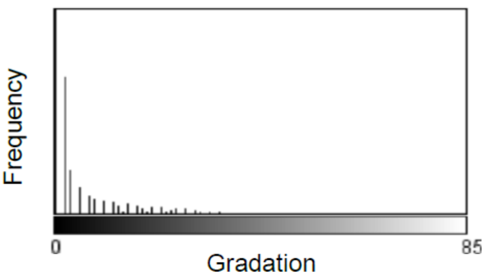

(**b**)

Figure S2. Fluorescence intensity histograms of GVs stained with BODIPY-HPC and BODIPY-**C**. The histograms were obtained from fluorescence images of GVs stained with BODIPY-HPC (**a**) and BODIPY-**C** (**b**) by Fiji software. The histograms support indistinguishability of analyzed fluorescence intensities between BODIPY-HPC and BODIPY-**C** in the current confocal microscopy observation.

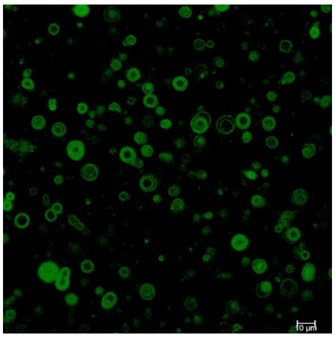

(**a**) BODIPY channel

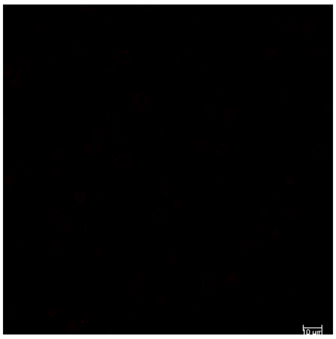

(**b**) Texas Red channel

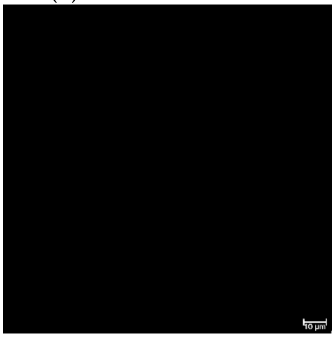

(**c**) BODIPY channel

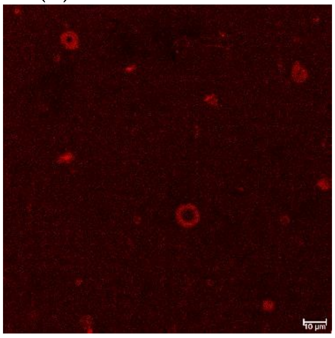

(**d**) Texas Red channel

Figure S3. Fluorescence compensation in confocal microscopy observation. Confocal microscopy images of GVs stained with BODIPY-HPC (**a**,**b**) or Texas Red-DNA (**c**,**d**). The leakage of fluorescence from another fluorescence channel was avoided to prevent the increases of the apparentcolocalization and of the average nMDP of each image.

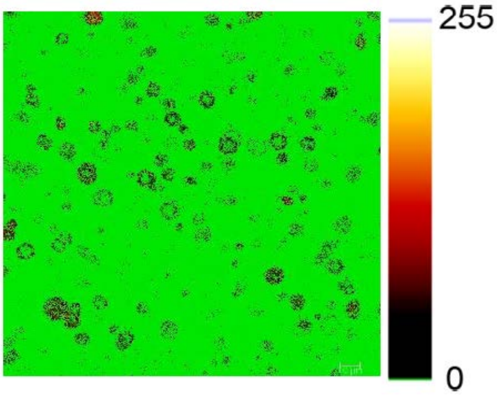

(**a**) BODIPY channel

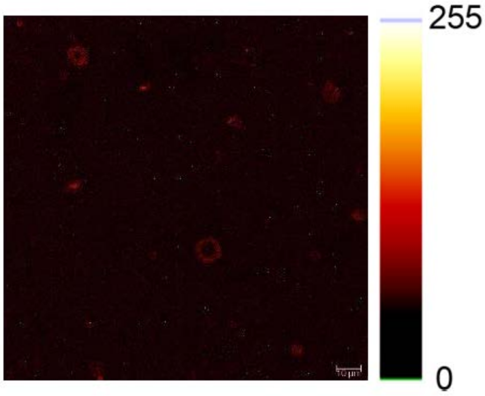

(**b**) Texas Red channel

Figure S4. Prevention of fluorescence saturation in confocal microscopy observation. The saturation of fluorescence must be prevented because it decreases validity of obtained nMDP value. Blue means saturation of the fluorescence intensity. The heatmap of GVs stained with BODIPY-HPC (**a**) was red-black, and that of Texas Red DNA (**b**) was red-black. No saturation was recognized in both fluorescence intensities.

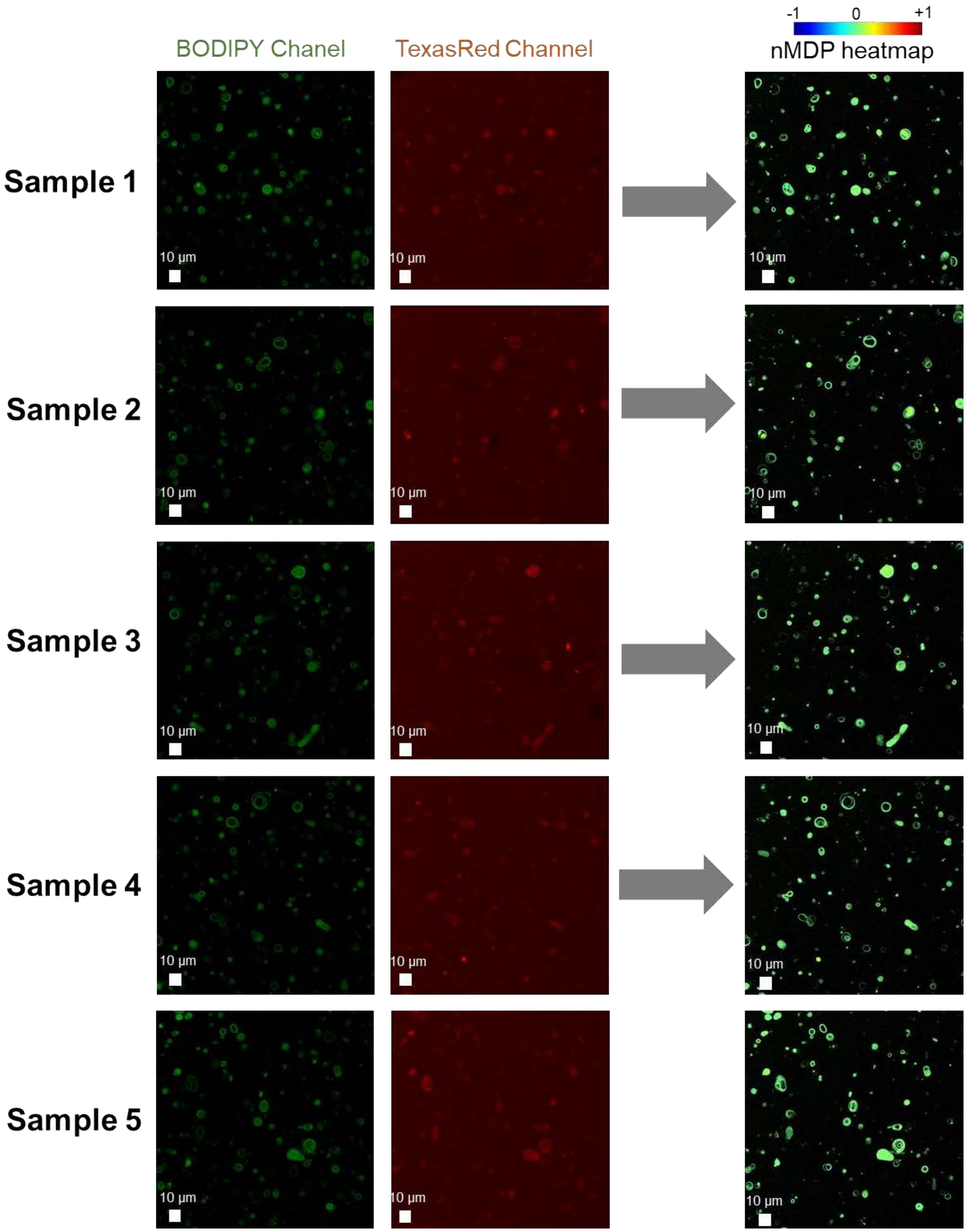
-1
0
+1
nMDP heatmap
BODIPY Chanel
TexasRed Channel
Sample 1
Sample 2
Sample 3
Sample 4
Sample 5
10 µm

(**a**)

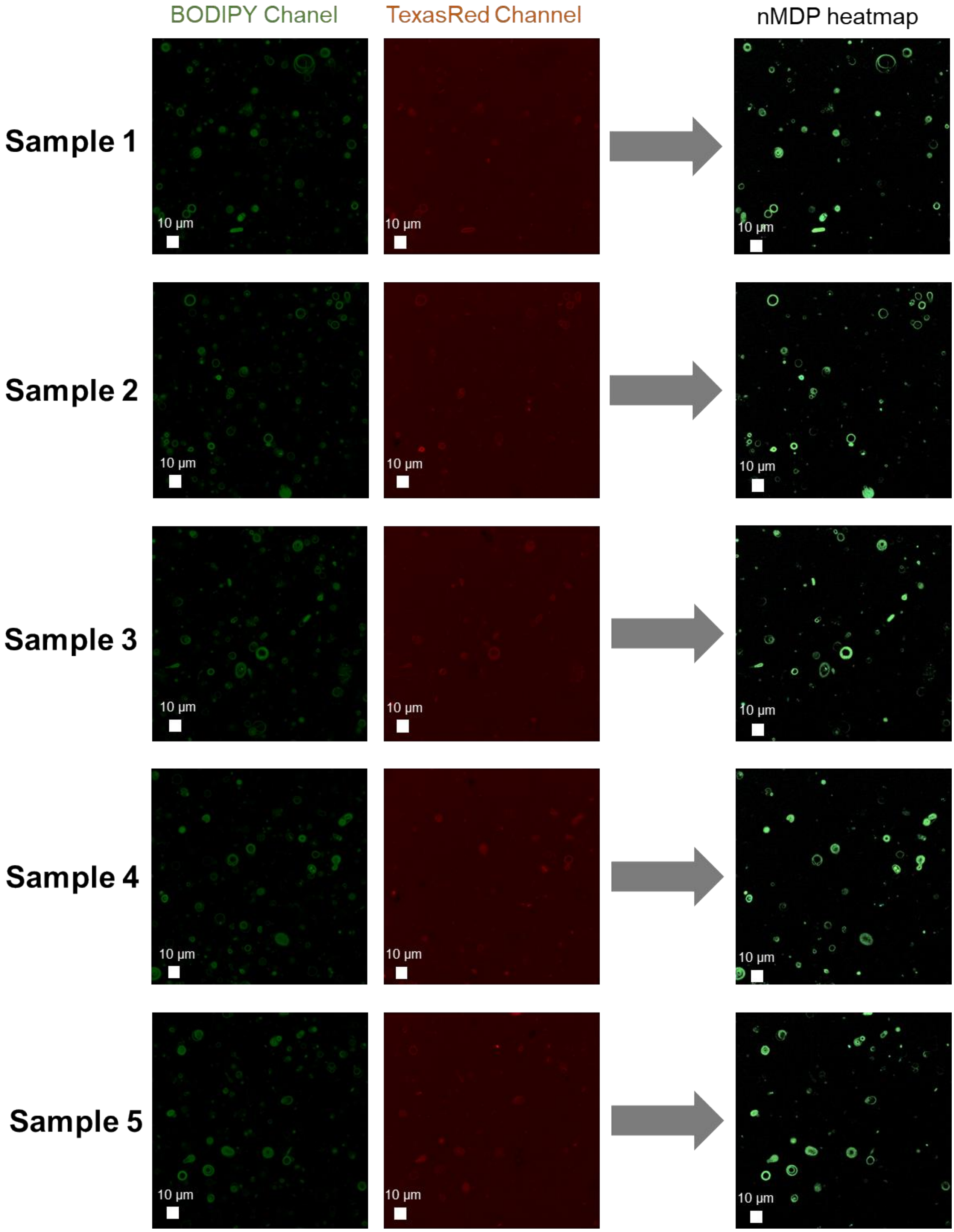

(**b**)

Figure S5. nMDP heatmaps for DNA-Texas Red and BODIPY-C (**a**) and DNA-Texas Red and BODIPY-HPC (**b**) originating the histogram in Figure 4. Since the resolution of the original images for nMDP calculation such as in Figure 4 is 2048 × 2048 pixel^2, whole pixel number of each nMDP map is 4,194,304.

Table S1. Indistinguishability of analyzed fluorescence intensities between BODIPY-HPC and BODIPY-C in current confocal microscopy observation. The average intensities were obtained from fluorescence images of GVs stained with BODIPY-HPC and BODIPY-**C** by Fiji software. Significant differences were not detected between intensities in confocal microscope images of BODIPY-HPC and BODIPY-**C** captured in five different sites in fields of view.

**BODIPY-HPC**	
Site 1	1.22
Site 2	1.19
Site 3	1.15
Site 4	1.02
Site 5	0.9
Average intensity	1.12

**BODIPY-C**	
Site 1	1.28
Site 2	1.18
Site 3	1.07
Site 4	1.01
Site 5	0.88
Average intensity	1.08

Table S2. Number of pixels calculated nMDP on each heat maps represented in Figure S5. Wholepixel number including non-calculated pixels is 4,194,304 (= 2048^2).

DNA-Texas Red and BODIPY-C

Heat map	nMDP-calculated pixels
#1	184,064
#2	154,162
#3	165,823
#4	168,115
#5	201,105
average	174,654

DNA-Texas Red and BODIPY-HPC

Heat map	nMDP-calculated pixels
#1	82,542
#2	68,800
#3	64,886
#4	79,829
#5	70,426
average	77,296

Table S3. Population ratio of pixels with magnitude of nMDP > −1.0 or nMDP < +0.1 on thenMDP heat map.

DNA-Texas Red and BODIPY-C

nMDP	**Popula tion rati o (%)*** #1	#2	#3	#4	#5	Averaged population ratio (%)	Standard Error	Averaged pixel number**
<−0.1	0.5585	0.6733	0.4487	0.3896	0.5575	0.53	±0.05	926
> 0.1	1.0757	1.7092	1.4148	0.6585	1.1641	1.20	±0.17	2095

DNA-Texas Red and BODIPY-HPC

nMDP	**Popula tion rati o (%)*** #1	#2	#3	#4	#5	Average of population ratio (%)	Standard Error	Average of pixel number**
<−0.1	0.4824	0.6831	0.5717	0.8356	0.8321	0.68	±0.07	525
> 0.1	0.2689	0.5741	0.6626	0.4735	0.1732	0.31	±0.09	239

* "Population ratio" is defined as the ratio of nMDP-calculated pixel number with magnitude ofnMDP < −0.1(or nMDP > 0.1), $n_{\pm 0.1}$, against whole number of nMDP-calculated pixels (Table S2), n_{whole}: $n_{\pm 0.1}/n_{\text{whole}} \times 100$ (%).

** "Average of pixel number" is product between the average of population ratio in this table and theaverage of whole number of nMDP-calculated pixels in Table S2. This number indicates that realnumber of pixels that fulfill the condition for one heat map.
